# Generation of two human control iPS cell lines (UCLi016-A and UCLi017-A) from healthy donors with no known ocular conditions

**DOI:** 10.1016/j.scr.2020.102113

**Published:** 2020-12

**Authors:** Cécile Méjécase, Philippa Harding, Hajrah Sarkar, Jonathan Eintracht, Dulce Lima Cunha, Lyes Toualbi, Mariya Moosajee

**Affiliations:** a UCL Institute of Ophthalmology, London, UK; b The Francis Crick Institute, London, UK; c Moorfields Eye Hospital NHS Foundation Trust, London, UK; d Great Ormond Street Hospital for Children NHS Foundation Trust, London, UK

## Abstract

Two human induced pluripotent stem cell (hiPSC) lines (UCLi016-A and UCLi017-A) were generated from fibroblast cells of 23- and 34-year-old healthy male donors with no known ocular conditions. Fibroblast cells were derived from skin biopsies and reprogrammed using integration free episomal reprogramming. The established iPSC lines were found to express pluripotency markers, exhibit differentiation potential *in vitro* and display a normal karyotype. These cell lines will act as a control lines for researchers studying ocular diseases.

## 1 Resource table

**Table:** 

Unique stem cell lines identifier	UCLi016-A UCLi017-A
Alternative names of stem cell lines	WT1 (UCLi016-A) WT2 (UCLi017-A)
Institution	UCL Institute of Ophthalmology
Contact information of distributor	Mariya Moosajee (m.moosajee@ucl.ac.uk)
Type of cell lines	iPSC
Origin	Human
Cell Source	Dermal fibroblasts
Clonality	Clonal
Method of reprogramming	Episomal plasmid
Multiline rationale	Controls
Gene modification	No
Type of modification	N/A
Associated disease	N/A
Gene/locus	N/A
Method of modification	N/A
Name of transgene or resistance	N/A
Inducible/constitutive system	N/A
Date archived/stock date	N/A
Cell line repository/bank	N/A
Ethical approval	12/LO/0489

## 2 Resource utility

Human induced pluripotent stem cell (hiPSCs) generated from two healthy male donors with no known ocular conditions using integration-free episomal reprogramming from fibroblasts will act as control lines for studying cellular models of ocular disease.

## 3 Resource details

hiPSCs provide a resource to investigate human conditions which would otherwise be inaccessible to study. Patient-derived iPSCs with known pathogenic mutations may elucidate the molecular basis of genetic ocular disorders through *in vitro* human cellular disease modelling. This knowledge will aid in understanding the phenotypic variation observed in patient cohorts, improving diagnosis and management, in addition to allowing development of novel treatments. In order to study misregulated gene and protein function in disease lines, it is necessary to have stable control lines derived from healthy individuals.

In this study, two iPSC lines were derived from the fibroblasts of the healthy male donors with no known ocular conditions (Table 1). These iPSC lines can be used as control lines for research into cellular models of ocular disease.

**Table 1:** Summary of lines.

iPSC line names	Abbreviation in figures	Gender	Age	Ethnicity	Genotype of locus	Disease
UCLi016-A	WT1	Male	28	Caucasian	N/A	N/A
UCLi017-A	WT2	Male	34	Caucasian	N/A	N/A

**Fig. 1:**
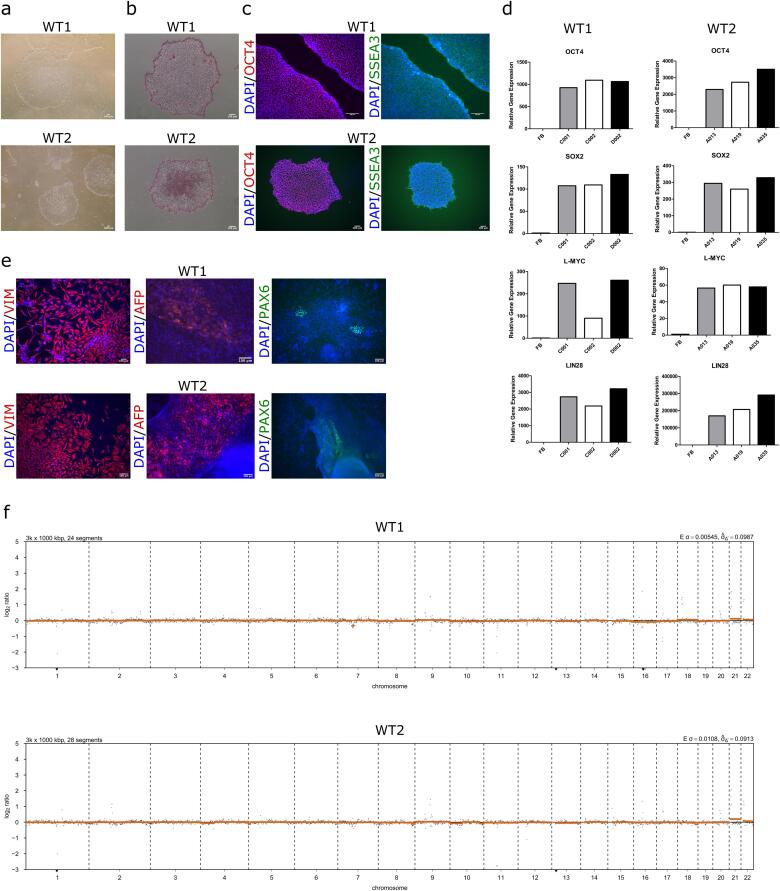
Characterisation of iPSC lines WT1 (UCLi016-A) and WT2 (UCLi017-A) generated from healthy dermal fibroblasts. (a) Brightfield images of healthy iPSC colonies. (b) Alkaline phosphatase activity in healthy iPSCs. (c) Immunofluorescent staining of cells expressing pluripotency markers SSEA3 (green) and OCT4 (red), with nuclear DAPI stain (blue). (d) Quantitative PCR analysis of stem cell markers OCT4, SOX2, L-MYC and LIN28. Glyceraldehyde 3-phosphate dehydrogenase (GAPDH), housekeeping gene. (e) Immunofluorescent staining of cells derived from embryoid body differentiation expressing markers of three germ layers: endoderm marker AFP (red), mesoderm marker Vimentin (VIM) (red) and ectoderm marker PAX6 (green). (f) Low-pass whole genome sequencing displaying normal male 46XY karyotypes.

With ethical approval, skin biopsies were taken and fibroblasts were derived. Fibroblasts were reprogrammed into iPSCs using non-integrating episomal plasmids encoding the reprogramming factors *OCT4*, *KLF4*, *SOX2*, *L-MYC* and *LIN28* as well as transient transcription enhancer *EBNA* (Table S1) (Parfitt et al., 2016). Embryonic stem cell-like colonies were picked, and three iPSC clones per line were expanded and characterised for pluripotency (Table 2). iPSC morphology was examined showing flat, compact colonies and cells with cobblestone appearance and large nuclei to cytoplasmic ratio (Fig. 1A). iPSCs were positively stained for alkaline phosphatase activity (Fig. 1B) and key pluripotency markers OCT4 and SSEA3 (Fig. 1C). Gene expression of pluripotency markers *OCT4*, *SOX2*, *L-MYC* and *LIN28* was validated using qRT-PCR analysis, which showed upregulation of these markers compared to fibroblast controls (Fig. 1D). *In vitro* differentiation ability after embryoid body formation showed positive staining for all three germ layers, using endoderm marker AFP, mesoderm marker Vimentin (VIM) and ectoderm marker PAX6 (Fig. 1E). Low-pass whole genome sequencing analysis of iPSCs revealed a normal male 46XY karyotypes (Fig. 1F). Genetic signature identity of fibroblasts and iPSCs was confirmed through STR analysis (submitted to journal). Absence of Mycoplasma was confirmed in iPSCs (Table S2).

**Table 2:** Characterization and validation.

Classification	Test	Result	Data
Morphology	Photography	Normal	Fig. 1 panel A
Phenotype	Qualitative analysis: Immunocytochemistry	Positive for pluripotency markers OCT4 and SSEA3	Fig. 1 panel C
	Qualitative analysis: Alkaline phosphatase activity	Visible activity	Fig. 1 panel B
	Quantitative analysis: qRT-PCR	Expression of *OCT4, SOX2, L-MYC* and *LIN28* in WT1 C002, WT1 C002, WT1 D002, WT2 A002, WT2 A019 and WT2 A035 clones and absence of expression in fibroblasts (FB)	Fig. 1 panel D
Genotype	Low-pass whole genome	46XY	Fig. 1 panel F
Identity	Microsatellite PCR (mPCR)	N/A	N/A
	STR analysis	16 STR analyzed, all matched	Submitted to journal
Mutation analysis	Sequencing	N/A	N/A
	Southern Blot OR WGS	N/A	N/A
Microbiology and virology	Mycoplasma	Mycoplasma testing by MycoAlert™ Mycoplasma Detection Kit (Lonza): Negative	Supplementary Table 2
Differentiation potential	Embryoid body formation	Positive for three germ layer markers: endoderm marker AFP, mesoderm marker Vimentin (VIM) and ectoderm marker PAX6	Fig. 1 panel E
Donor screening	HIV 1 + 2 Hepatitis B, Hepatitis C	N/A	N/A
Genotype additional info	Blood group genotyping	N/A	N/A
	HLA tissue typing	N/A	N/A

In conclusion, two wild-type (WT) control hiPSCs were generated from fibroblasts of healthy male donors with no known ocular conditions. These iPSC lines will be used as control lines for disease modelling, to aid in understanding molecular pathology of ocular disease, identifying therapeutic targets and drug screening.

## 4 Materials and methods

### 4.1 Fibroblast derivation and culture

Skin biopsies were placed in 400 μL digestion media (DMEM high glucose with pyruvate/glutamine (Gibco), 20% fetal bovine serum (FBS) (Gibco), 0.25% Collagenase I (Gibco), 0.05% DNase I (Gibco), penicillin/streptomycin (Gibco)), incubated overnight, then plated in derivation media (DMEM, 20% FBS, penicillin/streptomycin). Fibroblasts were cultured in fibroblast media (DMEM, 15% FBS, pen/Strep) and passaged with TrypLE Express (Gibco).

### 4.2 Fibroblast reprogramming and iPSC culture

1 × 10^6^ fibroblast cells were electroporated (1700 V, 20 ms, 1 pulse) with 1 μg of each episomal plasmid (Table S1) using the Neon Transfection System (Parfitt et al., 2016). Transfected cells were plated in fibroblast media with 0.5 mM sodium butyrate in a 0.1% gelatin-coated 100 mm dish for 7 days. Cells were dissociated with TrypLE Express and 200,000 cells plated into each well of a Matrigel-coated (Corning) 6-well plate in mTeSR Plus (Stemcell Technologies). Colonies were picked manually for the first 4 passages, then passaged using ReLeSR (Stemcell Technologies) at 70% confluency.

### 4.3 Alkaline phosphatase staining

Cells were stained using the StemAb Alkaline Phosphatase Staining Kit II (Reprocell).

### 4.4 Immunocytochemistry

Cells were fixed using 4% PFA for 20 min at 4 °C, permeabilized and blocked for 1 h using 10% normal goat serum (NGS) and 0.1% Triton X-100 in PBS at RT. Cells were incubated for 1 h at RT with primary antibodies diluted in 1% NGS (Table 3). Secondary antibodies and DAPI were added for 1 h at RT (Table 3). Cells were imaged using the EVOS M7000 Imaging System.

**Table 3:** Reagents details.

Antibodies used for immunocytochemistry			
	Antibody	Dilution	Company Cat # and RRID
Pluripotency Markers	Mouse anti-OCT4	1:100	Santa Cruz Biotechnology Cat# sc-5279, RRID:AB_628051
	Rat anti-SSEA3	1:50	Millipore Cat# MAB4303, RRID:AB_177628
Differentiation Markers	Mouse anti-AFP	1:300	Santa Cruz Biotechnology Cat# sc-51506, RRID:AB_626514
	Mouse anti-VIM	1:250	Santa Cruz Biotechnology Cat# sc-6260, RRID:AB_628437
	Rabbit anti-PAX6	1:100	Covance Cat# PRB-278P, RRID:AB_291612
Secondary antibodies	Goat anti-Mouse IgG (H + L) Cross-Adsorbed Secondary Antibody, Alexa Fluor 647	1:400	Thermo Fisher Scientific Cat# A-21235, RRID:AB_2535804
	Goat anti-Rat IgG (H + L) Highly Cross-Adsorbed Secondary Antibody, Alexa Fluor 488	1:400	Thermo Fisher Scientific Cat# A-11006, RRID:AB_2534074
	Goat anti-Rabbit IgG (H + L) Highly Cross-Adsorbed Secondary Antibody, Alexa Fluor 488	1:400	Thermo Fisher Scientific Cat# A32731, RRID:AB_2633280
	Goat anti-Mouse IgG (H + L) Cross-Adsorbed Secondary Antibody, Alexa Fluor 488	1:400	Thermo Fisher Scientific Cat# A-10011, RRID:AB_2534069

Primers			
	Target	Forward/Reverse primer (5′-3′)	
Pluripotency Markers (qRT-PCR)	OCT4	CCCCAGGGCCCCATTTTGGTACC/ ACCTCAGTTTGAATGCATGGGAGAGC	
	SOX2	TTCACATGTCCCAGCACTACCAGA/ TCACATGTGTGAGAGGGGCAGTGTGC	
	LIN28	AGCCATATGGTAGCCTCATGTCCGC/ TCAATTCTGTGCCTCCGGGAGCAGGGTAGG	
	L-MYC	GCGAACCCAAGACCCAGGCCTGCTCC/ CAGGGGGTCTGCTCGCACCGTGATG	
House-Keeping Genes (qRT-PCR)	GAPDH	ACAGTTGCCATGTAGACC/TTTTTGGTTGAGCACAGG	

### 4.5 qRT-PCR

RNA was extracted from cell pellets using RNeasy Mini Kit (Qiagen) and 1 μg of cDNA synthesised using SuperScript III First-Strand Synthesis kit (Invitrogen). qRT-PCR was performed using SYBR green mastermix (Applied Biosystems), run on the StepOne Plus RealTime PCR System (Thermo Fisher) using standard cycle conditions with designed primers (Sigma Aldrich) (Table 3) (Ye et al., 2012). The relative expression of each target gene was normalised to housekeeper *GAPDH* and compared to fibroblast expression using the comparative CT method.

### 4.6 Embryoid body mediated spontaneous *in vitro* differentiation

Embryoid bodies were formed by cell dissociation with ReLeSR and culturing in Aggrewell media (Stemcell Technologies) supplemented with 10 µM Y27632 (Abcam) for 7–10 days. Embryoid bodies were plated in 0.1% gelatin-coated plates for 11–15 days, where embryoid bodies attached and spontaneously differentiated. Cells were fixed and immunostained for AFP, Vimentin and PAX6 (Table 3).

### 4.7 Low-pass whole genome sequencing and STR analysis

DNA was extracted using QIAamp DNA Micro Kit (Qiagen). For low-pass WGS, libraries were produced using the Illumina DNA Prep library prep kit and sequenced on the Illumina HiSeq 4000 with paired 100 bp reads. After alignment, copy number estimation was performed using the QDNASeq package (Scheinin et al., 2014). Short Tandem Repeat (STR) profiling of 16 sites was obtained for iPSC and fibroblast lines with the Promega PowerPlex16HS system and was compared back to any available on commercial cell banks (such as ATCC).

### 4.8 Mycoplasma testing

Absence of Mycoplasma contamination was confirmed using MycoAlert^TM^ Mycoplasma Detection Kit (Lonza).

## Declaration of Competing Interest

The authors declare that they have no known competing financial interests or personal relationships that could have appeared to influence the work reported in this paper.
