# Supplementary material for: Generation of two human control iPS cell lines (UCLi016-A and UCLi017-A) from healthy donors with no known ocular conditions
Source: Stem Cell Res. 2020 Dec;49:102113. doi: 10.1016/j.scr.2020.102113 (PMC7768179; doi:10.1016/j.scr.2020.102113)
Supplement: Supplementary data 1 [file mmc1.docx]

**Supplementary files -** UCLi016-A and UCLi017-A
**Supplementary Table 1** - Addgene Episomal Vectors for Reprogramming.

| **Episomal Plasmid** | **Addgene ID#** | **Encodes** |
| --- | --- | --- |
| **pCXLE-hSK** | 27078 | SOX2 and KLF4 |
| **pCXLE-hUL** | 27080 | L-MYC and LIN28 |
| **pCXLE-hOCT3/4-shp53-F** | 27077 | OCT3/4 and shRNA against p53 |
| **pCXWB-EBNA1** | 37624 | transient EBNA-1 |

**Supplementary Table 2** – MycoAlert^TM^ Mycoplasma Detection Kit (Lonza) results for WT1 and WT2 lines. Ratio <1.2 – negative.

| **Sample** | **Reading A** | **Reading B** | **Ratio** |
| --- | --- | --- | --- |
| **Water Control** | 12606 | 1875 | 0.148739 |
| **WT1** | 7949 | 4148 | 0.521827 |
| **WT2** | 66790 | 28780 | 0.430903 |
